# An all-in-one electrochromic neuromorphic display

**DOI:** 10.1093/nsr/nwaf515

**Published:** 2025-11-22

**Authors:** Wei Ma

**Affiliations:** State Key Laboratory for Mechanical Behavior of Materials, Xi’an Jiaotong University, China

**Figure 1.**
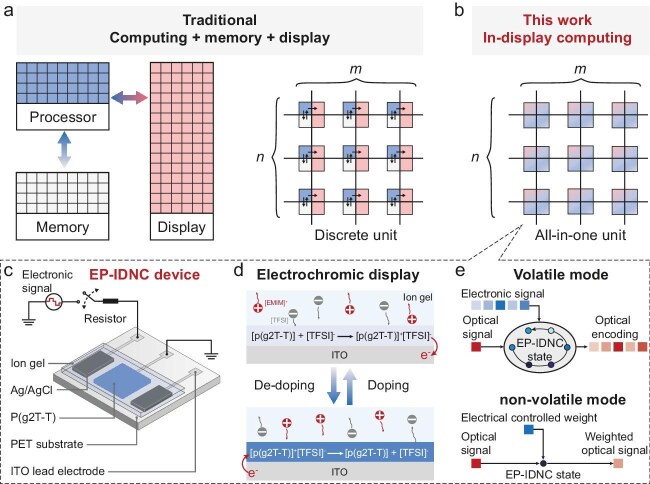
(a, b) Schematics comparing traditional display and computing technology with electrochromic neuromorphic displays. (c) Structure of the reconfigurable multi-terminal EP-IDNC device. (d) Operating mechanism of the EP-IDNC as a display unit. (e) Implementation of various computing functions via volatile and nonvolatile modes in the EP-IDNC, in which electrically controlled transmittance serves as synaptic weight.

The rapid expansion of intelligent applications on edge devices is driving demand for high-efficiency computing systems that deliver ultra-low latency, minimal energy consumption and seamless human–machine interaction [1]. Conventional edge devices, based on von Neumann architectures with physically separated memory, processing and display units, incur substantial energy and latency penalties from frequent data transfers and repeated analog-to-digital conversions (Fig. 1a). While neuromorphic computing has emerged as a promising alternative by using in-memory processing to reduce data movement [2,3], most existing neuromorphic devices lack built-in visualization capabilities, restricting real-time interactive feedback.

Recent advances in neuromorphic light-emitting devices have aimed to bridge this gap by converting electrical signals into dynamic visible emissions, enabling direct visualization of computational states [1]. However, these systems often lack several essential neuromorphic capabilities: (i) multi-terminal operability, required for the dendritic integration of diverse input signals; (ii) reconfigurability, to dynamically switch between synaptic functions such as short-term and long-term plasticity; and (iii) stable nonvolatile memory, which is critical for retaining learned weights and supporting energy-efficient inference. The absence of these features impedes the implementation of sophisticated, brain-inspired computing. As a result, integrating in-memory computing, multi-terminal processing and real-time visualization into a single reconfigurable platform remains a significant and unresolved challenge.

To overcome these limitations, Dai and colleagues [4] developed an electrically programmable in-display neuromorphic computing (EP-IDNC) device that integrates memory, processing and display into a unified organic electrochromic platform (Fig. 1b and c). While electrochromism has been conventionally employed in smart windows and non-emissive displays, this work breaks new ground by creatively repurposing the electrochromic effect for neuromorphic computing. Through controlled electrochemical doping/dedoping and ion-modulated optical modulation, the device successfully emulates essential synaptic functionalities, including both short- and long-term plasticity, multi-terminal signal integration and spatio-temporal encoding. A particular innovation lies in its electrically tunable optical transmittance, which functions simultaneously as a programmable synaptic weight and an intuitive visual output, allowing computational results to be directly observed in real time (Fig. 1d and e). The authors validated the EP-IDNC concept with a prototype array performing real-time image noise reduction and motion tracking, and further demonstrated its practical utility through a car steering reminder system.

This study marks a notable advance in edge neuromorphic technology, establishing a new paradigm for neuromorphic displays and paving the way for highly integrated, energy-efficient and interactive edge Artificial Intelligence (AI) systems. The technology holds significant promise for applications in augmented reality, wearable electronics and autonomous systems. More broadly, it provides the foundation for a new category of intelligent displays that are not only responsive, but also incorporate embedded neuromorphic processing—representing a major step forward in the development of intelligent edge devices. While the current prototype shows promising functionality, its cycling endurance and switching speed still require further improvement to meet the stringent demands of real-world edge applications. Future iterations could explore optimized device architectures and a broader spectrum of electrochromic materials—such as inorganic transition-metal oxides (e.g. WO₃, NiO) and other organic semiconductors with tailored energy levels—to extend the operational wavelength range, enhance stability and boost overall performance.
